# Supplementary material for: Ectodysplasin overexpression reveals spatiotemporally dynamic tooth formation competency in stickleback and zebrafish
Source: Development. 2025 Sep 26;152(18):dev204907. doi: 10.1242/dev.204907 (PMC12516321; doi:10.1242/dev.204907)
Supplement: Supplementary information [file develop-152-204907-s1.pdf]

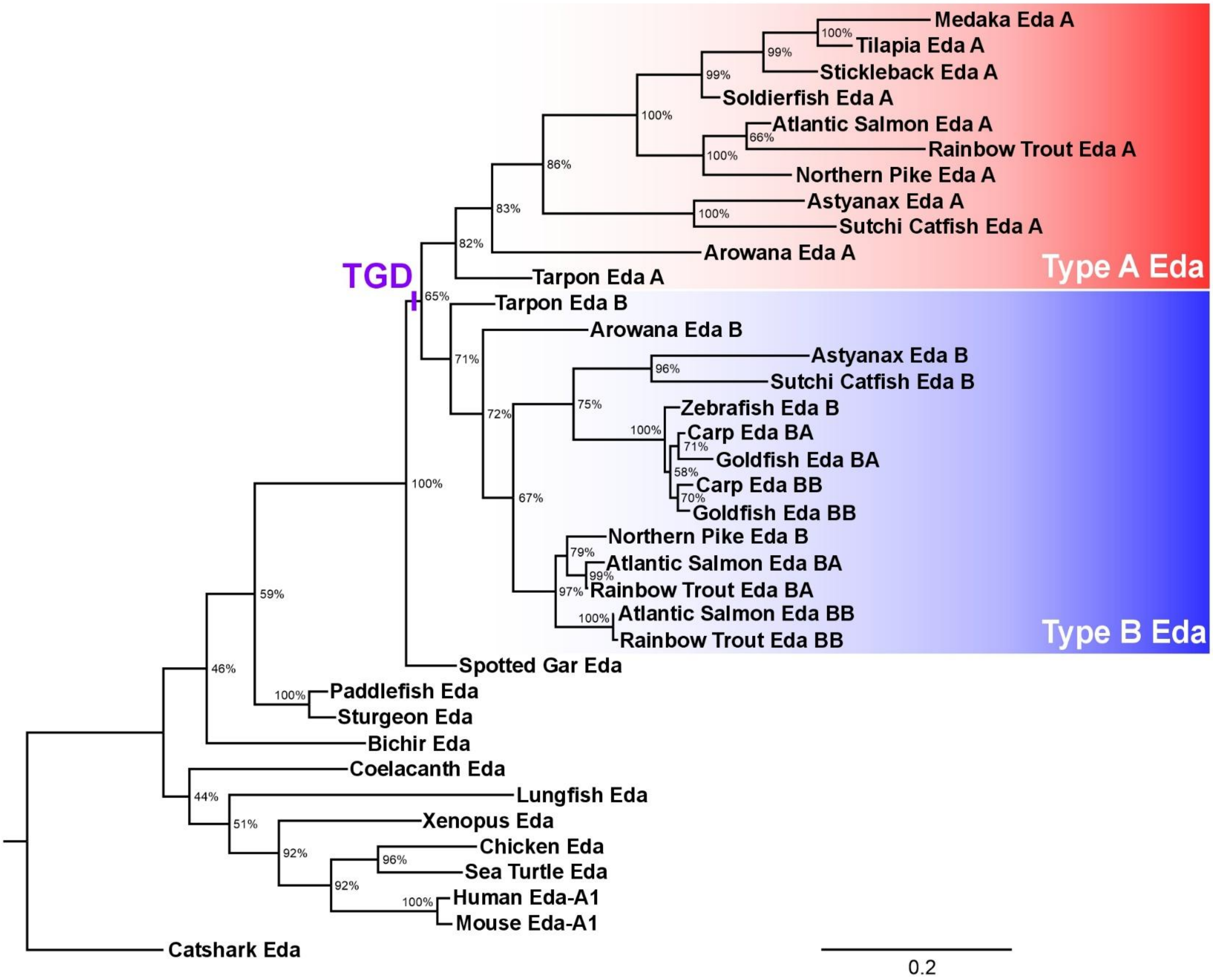

Fig. S1. Phylogenetic tree of Eda amino acid sequences in jawed vertebrates. A Maximum Likelihood reconstruction based on an alignment of 37 Eda sequences from selected species. See Table S2 for accession numbers of the sequences included. A 100 replicate bootstrap test was performed, all resulting values are shown near each node. Catshark Eda was selected as the outgroup. Of note: stickleback and zebrafish have retained opposite paralogs (A and B, respectively). Following the Teleost Genome Duplication (TGD), some groups appear to have further duplicated and/or lost one *Eda* paralog: two B group *Eda* genes were detected in two separate clades, carp+goldfish and salmonids; the salmonids assessed here thus uniquely appear to retain three *Eda* genes, one A and two B paralogs, while carp and goldfish have two *Eda* genes, both of which are B paralogs. See methods for aa alignment and tree-building parameters.

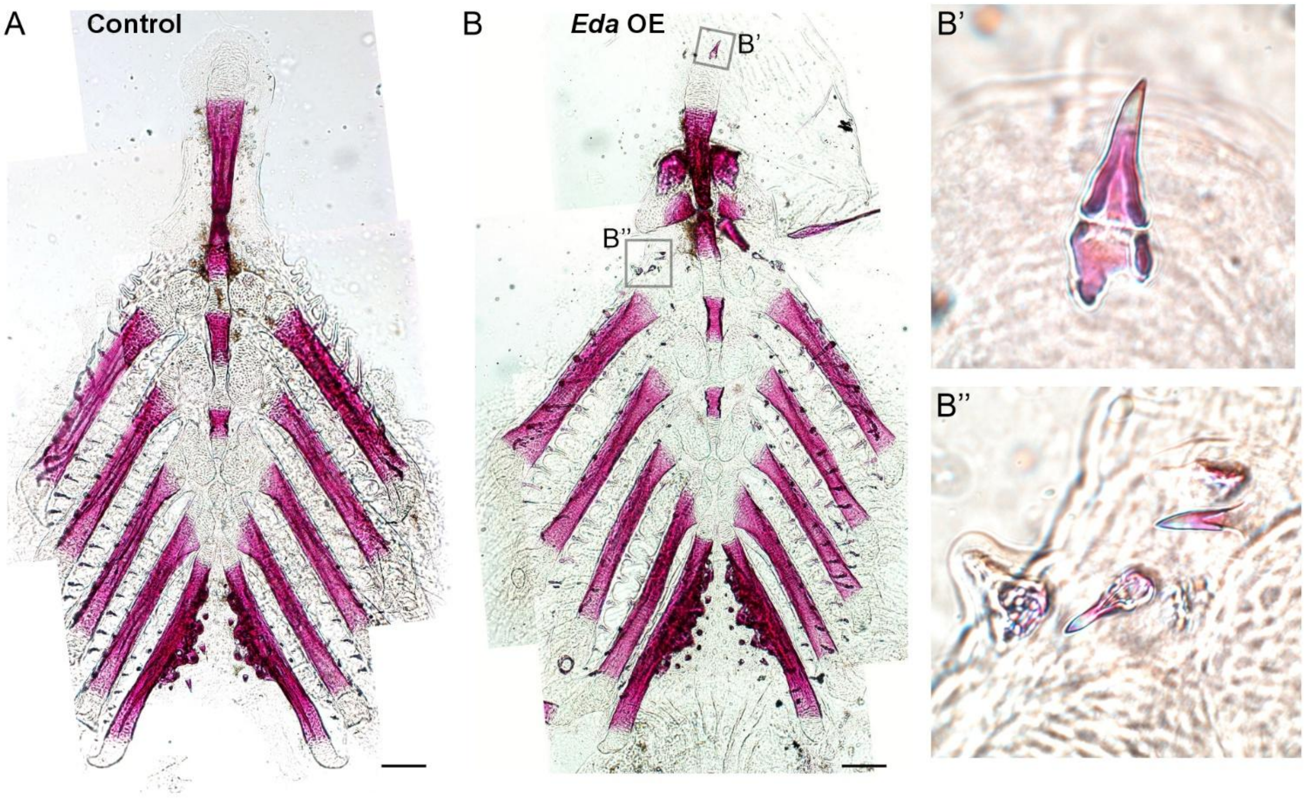

Fig. S2. Ectopic pharyngeal teeth resulting from larval *Eda* overexpression. Alizarin Red-stained pharyngeal preps from control (A) and *Eda* OE (B) fish that underwent the larval OE treatment. Insets are indicated in the *Eda* OE treatment (B' and B") to show detail of ectopic teeth in the *Eda* OE condition. Scale bars=200 μm.

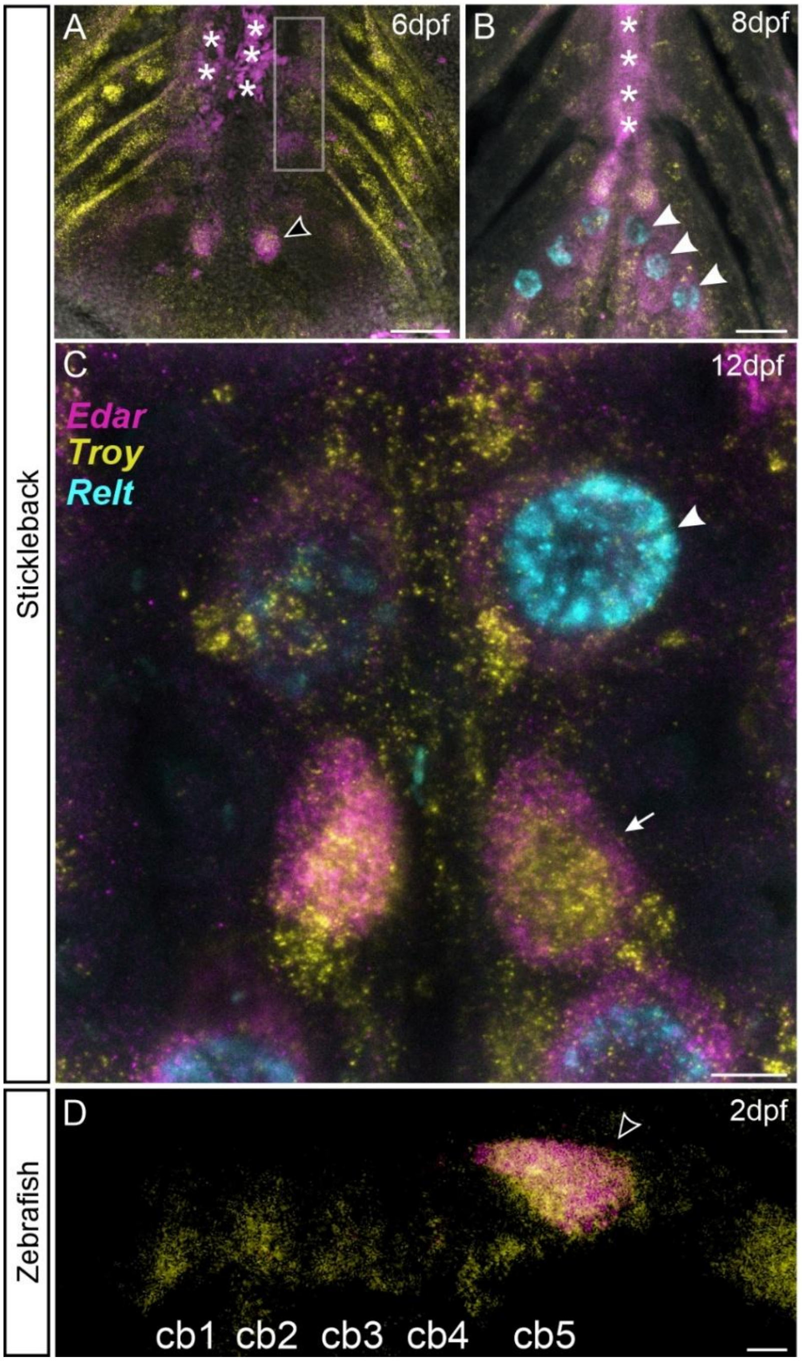

Fig. S3. *Edar*, *Troy*, and *Relt* expression in the stickleback pharynx. (A-C) Dorsal views (anterior to top) of TNFR HCR on dissected stickleback pharynx preparations. *Edar* and *Troy* transcripts were detected in stickleback pioneer teeth at 6 dpf (black arrowhead) and non-dental tissues presaging tooth formation competency (gray box). *Relt* is not present at 6 dpf and was only detected in ameloblasts during tooth differentiation at 8 and 12 dpf (arrowheads). White arrow marks a cap-stage tooth germ at 12 dpf. White asterisks mark autofluorescence from blood cells trapped near the heart. (D) sagittal Z slice (anterior to left) HCR of *edar* and *troy* in zebrafish show expression in the pioneer tooth at 2 dpf (black arrowhead) similar to the distribution seen in stickleback. *troy* expression is additionally detected throughout the ventral pharynx (cb1-4) but *edar*expression was not detected as it was in sticklebacks. Scale bars in A,B=100 μm, C,D=20 μm.

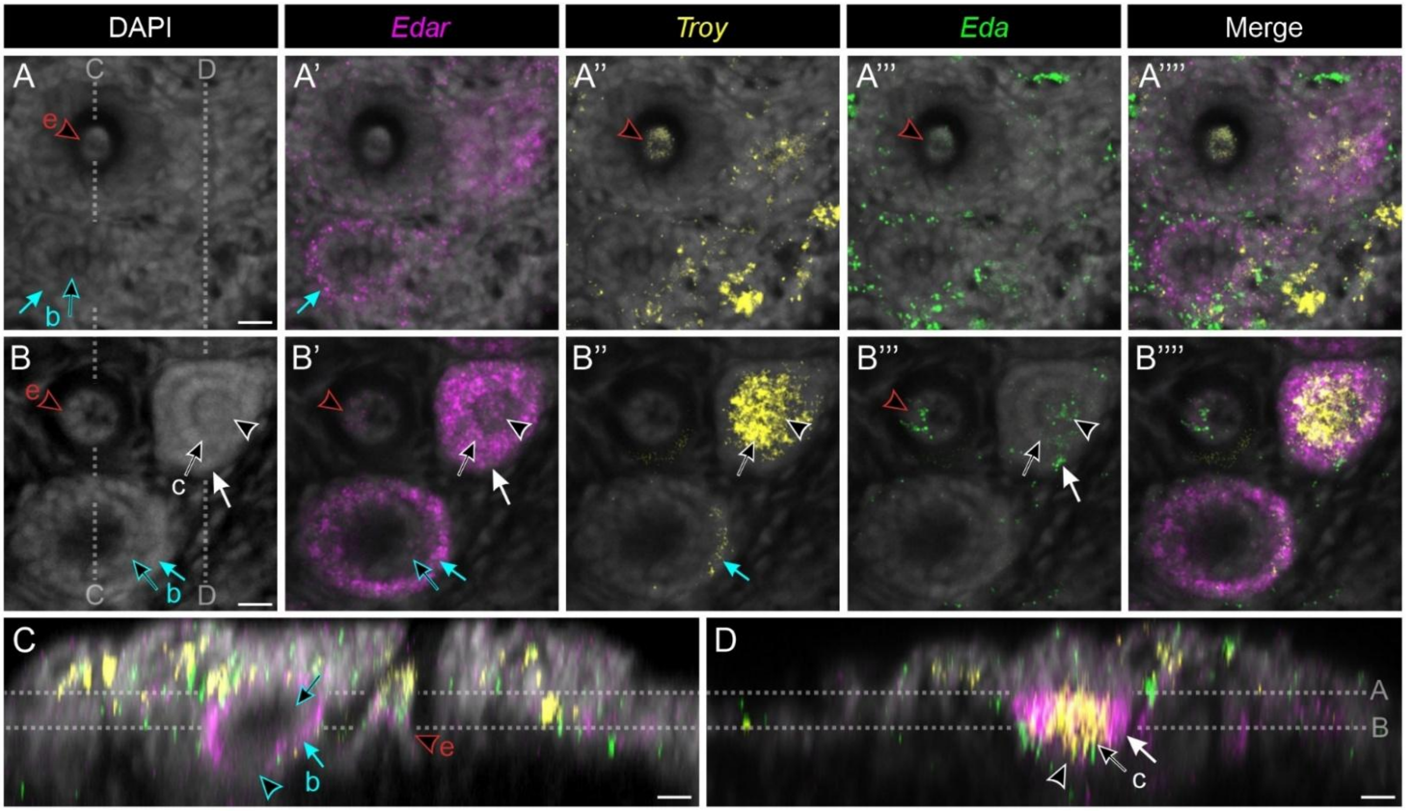

Fig. S4. ***Edar*, *Troy*, and *Eda* expression within and surrounding tooth germs.** A cluster of three tooth organs on dorsal tooth plate 2 (DTP2) at 18 dpf are shown as example to further compare tooth germ expression at higher resolution. (A,B) optical sections showing a shallow (A) and deep (B) plane of section reveal dental mesenchyme of an erupted tooth (“e” in A-C, red and black arrowheads), bell-stage tooth germ (“b” in A-C) inner dental epithelium (black and cyan arrows) and outer dental epithelium (cyan arrows), and cap-stage (“c” in A,B,D) mesenchyme (black and white arrowhead), inner dental epithelium (black and white arrow), and outer dental epithelium (white arrow). All tissues are labeled in the DAPI panels, while the subsequent panels show arrows or arrowheads only if transcripts were detected in that tissue. Merged panels are shown without markup. Gray dotted lines in A and B labeled “C” and “D” indicate the plane of the orthogonal views in panels C and D. We detected *Edar, Troy,*and *Eda*within and/or surrounding tooth organs of most developmental stages. Cap stage tooth germs (“c”) demonstrated the most widespread expression of all three genes, where *Edar* was detected in both inner and outer dental epithelium (B’, black and white arrows) as well as dental mesenchyme (B’, black arrowhead), *Troy*most strongly marked inner dental epithelium (B’’, black arrow) and mesenchyme (B’’, black and white arrowhead), and *Eda* sparsely marked inner and outer dental epithelium and mesenchyme (B’’’, black arrowhead). Bell-stage tooth germs (“b”) demonstrated more restricted expression, with *Edar* mainly detected in the outer dental epithelium (B’, cyan arrow) with some limited *Troy* signal in the same tissue (B’’, cyan arrow), and little signal seen in mesenchyme (C, cyan and black arrowhead). Erupted tooth mesenchyme (“e”) expressed *Troy*apically (A’’, red and black arrowhead) while demonstrating limited *Edar* and *Eda* signal at a deeper level (B’,B’’’, black arrowheads). (C,D) Orthogonal composite views confirmed this general scheme. Labeling and arrow scheme same as above, all tissues are marked with arrows regardless of detected expression. Scale bars=10 μm.

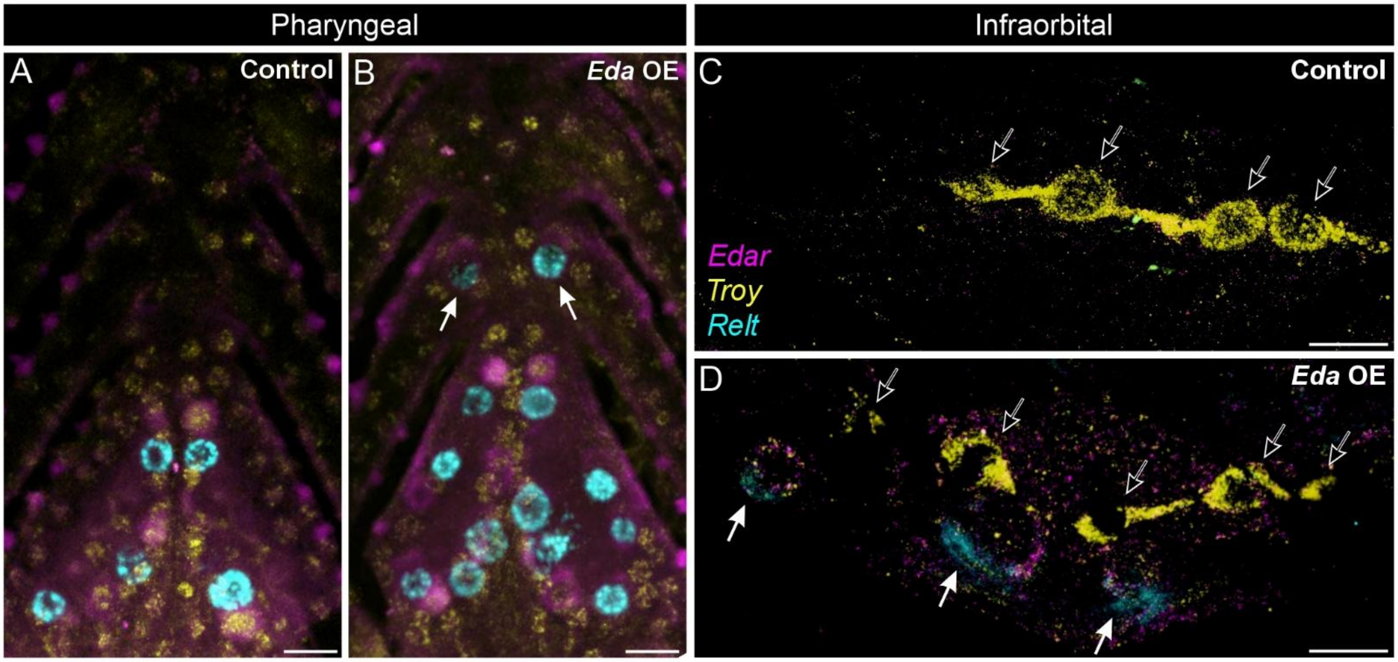

Fig. S5. *Edar*, *Troy*, and *Relt* expression are found in ectopic tooth germs. HCR was performed on control and *Eda* OE fish to assay TNFR expression in nascent ectopic teeth and surrounding tissues. (A,B) Fish heat shocked from 6-8 dpf (1x per day, 3 total heat shocks) were fixed at 14 dpf and subjected to HCR for *Edar*, *Troy*, and *Relt* (shown in magenta, yellow, and cyan, respectively). Anterior to top. Ectopic pharyngeal teeth (white arrows in B) display TNFR expression similar to endogenous teeth in the control condition (e.g. in panel A). (C,D) Fish heat shocked from 16-24 dpf (2x per day, 16 total heat shocks) were fixed at 30 dpf and subjected to HCR. Anterior to left. Ectopic face teeth (white arrows in D) also display TNFR expression similar to endogenous teeth, though at the stage assayed here, *Relt* expression appears more restricted than in endogenous teeth. *Troy* expression in neuromasts (black arrows) appeared typical in the control condition but displayed some disturbances (*Troy*-negativeregions) in the *Eda*OE condition, especially those neuromasts that were abutted by an ectopic tooth. We also noted a generally non-specific increase in *Edar* expression throughout the *Eda* OE samples. Scale bars=100 μm.

Development • Supplementary information

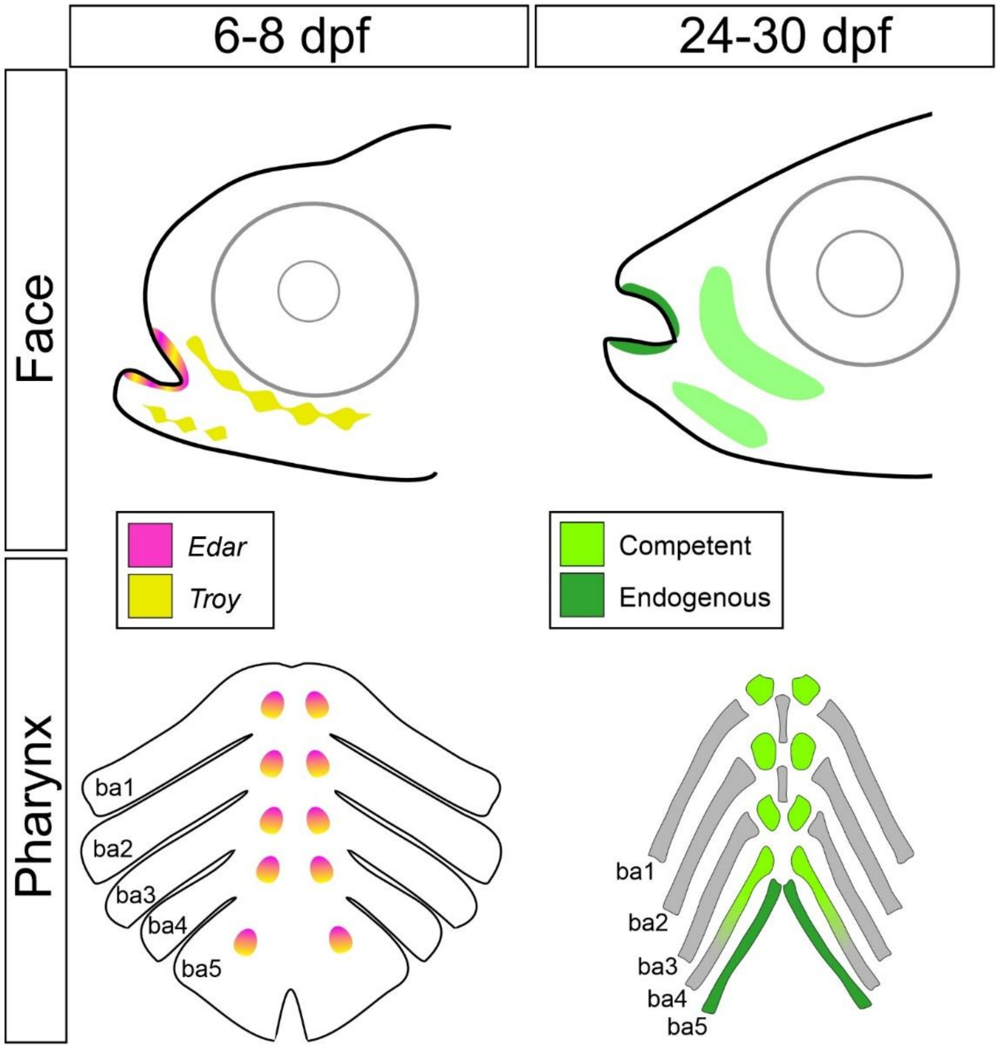

Fig. S6. Summary model for tooth formation competency and receptor expression for the pharynx and face in stickleback fish. At 6-8 dpf, *Troy* is found both orally and in nascent neuromasts, while *Edar*is found only orally. In the pharynx, *Edar* and *Troy* expression domains mark the ventral pharynx, left and right of the midline at each branchial arch (ba), including the endogenous tooth domain at ba5 (future ceratobranchial 5). At 24-30 dpf, tooth formation competency occurs endogenously in the mouth, but ectopically within and surrounding the facial neuromast fields. Within the pharynx, tooth formation endogenously occurs at ba5, but can ectopically occur at ba1-4.

Table S1. Threespine stickleback (*Gasterosteus aculeatus*) staging table and major developmental events by age in days when reared at 18-19° C. Summarized from Swarup, 1958, and Ellis *et al.*, 2016.

Day (dpf)	Key Developmental Events	Endogenous Tooth Events	Dental competency in response to Eda
Day 0	Stage 1. (0 h) Egg is fertilized.<br>Stage 2. (~1 ¾ h) Contractions accumulate cytoplast at animal pole.<br>Stage 3. (~2 ½ h) A cleavage furrow forms and divides the cell into two blastomeres.<br>Stages 3-7. (~3-4 h) Cell division continues. The embryo increases from 2 cells to 32 cells.<br>Stage 8-9. (~6-10 h) Cell divisions continue with both horizontal and vertical cleavage furrows. The periblast (a syncytial layer) forms between the yolk and morula.<br>Stage 10. (~15 h) Blastula formation starts with the morula flattening. A subgerminal cavity forms and the blastodisc goes from radially symmetrical to bilaterally symmetrical which defines the prospective anterior and posterior of the embryo.<br>Stage 11. (~22 h) Cells of the thicker sector of the blastodisc begin to invaginate marking the start of gastrulation.		
Day 1	Stage 12. (~26 h) Invagination extends laterally around the blastoderm until all periphery is invaginated. The germ ring forms.<br>Stage 13. (~30 h) Germ-ring expands out towards the equator of the egg.<br>Stage 14. (~36 h) Germ-ring continues to move past the equator (~50% epiboly) and blastoderm coverage increases. Neurulation begins.<br>Stage 15. (~42 h) Germ-ring continues moving until the blastopore is small (80-90% epiboly). There is a subdivision of the brain plate (fore-, mid-, and hind-brain) and embryo narrows/elongates.		
Day 2	Stage 16. (~50 h) Blastopore closes and optic lobes appear from lateral outgrowth of the fore-brain. Neurulation is complete; development of the nervous system results in embryo thickening.<br>Stage 17. (~60 h) Embryo is elevated from the yolk. First somites form.<br>Stage 18. (~70 h) Further differentiation of the head occurs, including optic vesicles into cups, dorsomedial furrow in the brain develops, optic capsules are visible and pericardium appears. Somite number continues to increase and Kupffer's vesicles become visible.		
Day 3	Stage 19. (~88 h) Brain enlarges and constriction forms between mid- and hind-brain. The heart becomes visible, starts beating.		
Day 4	Stage 20. (~106 h) Eyes become prominent with pigmentation forming in the peripheral margins. Ventricles in the midbrain have closed and the tail occasionally moves.		Eb1 pharyngeal domain becomes competent.

Development • Supplementary information

Day 5	Stage 21. (~130 h) The hind-brain ventricle has now closed and division of the heart chambers has occurred. Blood circulation begins.		Pharynx becomes competent in hb1 and cb4 domains with single Eda pulses. This is the sole dpf where 2% of fish gained a tooth at hb2 with a single pulse.
Day 6	Stage 22. (~144 h) Pectoral fins are visible and melanophores cover the entire eye and body progressing onto the yolk. Morphology of the head changes bringing the optic capsule more anteriorly. Pigmentation has also expanded on the eyes and body. Stage 23. (~156 h) Ventricles fully close and head is fully formed. The heart enlarges and circulation with the yolk is complete. Blood travels through the body to the yolk.	Pioneer tooth germs begin differentiating on ventral pharyngeal tooth plate (VTP) and the second (larger) dorsal pharyngeal tooth plate (DTP2).	Hb3 competency following a single Eda pulse present at a low level (2% of fish formed a tooth). Face becomes competent in the io domain with repeated Eda pulses.
Day 7	Stage 24. (~168 h) Mouth is formed, eye cup is complete. Fins vibrate at high frequency and the tail moves often.		Competency remains present at hb1, hb3, cb4, and eb1.
Day 8	Stage 25. (~192 h) Embryo hatches, transitioning to a larva, which is about 3 mm in total length.	VTP and DTP pioneer teeth begin ossification.	Competency remains present at hb1, cb4, and eb1.
Day 9	Stage 26. Larva is 3mm in length. The mouth is open and pectoral fins are functioning. Larva will lay on its side due to yolk and lack of an inflated swim bladder but can occasionally swim swiftly and resettle.		Pharyngeal competency remains only at the eb1 domain with single Eda pulses, and bh, hb1, hb2, hb3, and cb4 domains with multiple Eda pulses.
Day 10	Stage 27. Larva is about 4mm. Half of the yolk is absorbed and it can swim. The head straightens allowing the heart to unfold.	Pioneer tooth germs begin differentiating on DTP1.	Pharynx same as 9 dpf. The ventral dentary is now competent under multiple Eda pulses.
Day 11		Pioneer tooth germs begin differentiating on the dentary and premaxilla. All tooth fields are now specified.	
Day 12	Stage 28. Larva is about 6mm. All of the yolk is absorbed and the jaws develop so the mouth is functioning. Gill covers develop and the swim bladder is visible.	DTP1 pioneer teeth begin ossification.	
Day 13-16	Stage 29. Larva is about 8 mm. The continuous fin fold begins to divide. Pelvic spine forms and snout elongates.	Pioneer teeth are ossified on the dentary and premaxilla.	By day 14, the pharynx loses competence to respond to single Eda pulse.
Day 17-24	Stage 30. Larva is about 8.5-9.5mm. Fins and spines mature so that the dorsal and anal fins are triangular. Fin rays and dorsal spines are visible.		

Development • Supplementary information

Table S2. Accession numbers and genome assembly information for sequences used to reconstruct Eda amino acid sequence similarity in Fig. S1. Common names, Latin names, protein names, accession numbers, and the assembly from which each coding region was derived are listed in Fig. S1 (See electronic spreadsheet file downloadable from the Development website).

Available for download at
https://journals.biologists.com/dev/article-lookup/doi/10.1242/dev.204907#supplementary-data
