# Supplementary material for: Ectodysplasin overexpression reveals spatiotemporally dynamic tooth formation competency in stickleback and zebrafish
Source: Development. 2025 Sep 26;152(18):dev204907. doi: 10.1242/dev.204907 (PMC12516321; doi:10.1242/dev.204907)
Supplement: Supplementary file 1 [file develop-152-204907_review_history.pdf]

Ectodysplasin overexpression reveals spatiotemporally dynamic tooth formation competency in stickleback and zebrafish

Zoe Z. Chen, Sujanya N. Narayanan, Lena M. Stagliano, Peter Q. Huynh, Shivani Sundaram, Emma J. Mackey, Craig T. Miller and Tyler A. Square

DOI: 10.1242/dev.204907

Editor: Steve Wilson

Review timeline

Original submission: 1 May 2025
Editorial decision: 18 June 2025
First revision received: 18 July 2025
Accepted: 21 August 2025

Original submission

First decision letter

MS ID#: dev.204907

MS Title: Ectodysplasin overexpression reveals spatiotemporally dynamic tooth formation competency in stickleback and zebrafish

Authors: Zoe Z. Chen; Sujanya N. Narayanan; Lena M. Stagliano; Peter Q. Huynh; Shivani Sundaram; Emma J. Mackey; Craig T. Miller; Tyler A. Square
Article Type: Research Article

Dear Tyler,

I have now received all the referees' reports on the above manuscript, and have reached a decision. The referees' comments are appended below, or you can access them online: please go to:

As you will see, the referees express considerable interest in your work, but have some significant criticisms and suggestions for improving your manuscript. If you are able to revise the manuscript along the lines suggested, I will be happy receive a revised version of the manuscript. Please also note that Development will normally permit only one round of major revision. If it would be helpful, you are welcome to contact us to discuss your revision in greater detail. Please send us a point-by-point response indicating your plans for addressing the referees' comments, and we will look over this and provide further guidance.

Please attend to all of the reviewers' comments and ensure that you clearly highlight all changes made in the revised manuscript. Please avoid using 'Tracked changes' in Word files as these are lost in PDF conversion. I should be grateful if you would also provide a point-by-point response detailing how you have dealt with the points raised by the reviewers in the 'Response to Reviewers' box. If you do not agree with any of their criticisms or suggestions please explain clearly why this is so.

Reviewer 1

This manuscript presents a well-executed study exploring how ectopic overexpression of Ectodysplasin in transgenic sticklebacks and zebrafish can reveal latent tooth-forming competency

© 2025. Published by The Company of Biologists under the terms of the Creative Commons Attribution License (https://creativecommons.org/licenses/by/4.0/).

across multiple cranial and pharyngeal domains. The authors employ temporally controlled heat shock-induced Eda overexpression and demonstrate that ectopic tooth formation is highly spatiotemporally regulated, with pharyngeal teeth exhibiting regenerative potential, while facial teeth do not.

The manuscript is well structured, and includes all necessary details to follow the authors reasoning and experimental pipeline. I did enjoy reading the manuscript. My comments are very minor:

1. While the study identifies Edar and Troy expression patterns coincident with ectopic tooth formation, functional validation of receptor involvement is lacking. I recommend that the authors acknowledge this as a limitation or suggest it as a direction for future research in their discussion.

2. The manuscript presents a complex timeline of tooth formation competency without sufficient clarity on the specific developmental windows involved. A clearer presentation of the timing and anatomical specificity would enhance reader understanding.

3. The claim that ectopic pharyngeal teeth regenerate autonomously is intriguing but lacks direct evidence of self-renewal without external Eda input. Future studies should use lineage tracing or long-term live imaging to verify that these tooth fields can sustain multiple cycles of tooth replacement. Additionally, pulse-chase experiments or single-cell transcriptomics could provide insights into the cellular mechanisms underlying this regeneration.

Reviewer 2

SUMMARY OF THE ADVANCE MADE IN THIS PAPER AND ITS POTENTIAL SIGNIFICANCE TO THE FIELD

The Chen et al is an interesting exploration of how overexpressing Ectodysplasin (Eda) increases tooth number in sticklebacks and zebrafish. What differentiates this study form the previous ones is the construction and use of heat shock-inducible Eda, thereby allowing temporal regulation of Eda overexpression. The results provide a nuanced view into the effects of Eda, most notably how multiple treatments increase the formation of extra teeth in different locations. These suggest sensitivity of tooth making 'potential' being different in different locations of the fish body, many having evolutionary explanations. The analyses and images are high quality and I have no general reservations about the study.

SUGGESTIONS TO AUTHORS

1) It would make the paper stronger if there was a bit more explanation why sticklebacks and zebrafish should be studied in this manner (and not only one of the species). I am not questioning this approach, but as a narrative the reason for the use of these two taxa is left a bit for the imagination of the reader. Yes, zebrafish is the model species, and sticklebacks have more teeth so it is more interesting in this context, or something like that.

2) Presumably the repeated heat shock experiments (very nice Fig 5) increased the expression levels of Eda and availability of the EDA protein. Although measuring expression levels would be difficult in these kind of samples, the Fig 8 expression analyses makes one wonder how some of those would have changed in some of the treated samples (e.g. Fig 8E). I'll leave it for the authors to decide if any additional expression analysis is needed. Presumably the expression domains of Edar and Troy expand before the teeth become visible in the treated samples?

3) Line 93 "Provocatively, the ectopic teeth that arose in these experiments were often morphologically discontiguous with endogenous tooth fields,.."
Comment: Not sure what is provocative here. Intriguingly?

4) Line 459: "...while Osr2 loss-of-function has been shown to expand the endogenous molar tooth field in mice.."
Comment: reference?

© 2025. Published by The Company of Biologists under the terms of the Creative Commons Attribution License (https://creativecommons.org/licenses/by/4.0/).

5) 'Face teeth' is used to describe the ectopic teeth on the stickleback snout. This sounds a bit strange, and one wonders whether 'facial teeth' would be any better. I'll leave it for the authors to decide the nomenclature.

6) There is a reference to Fig. S5 in the text.

Reviewer 3

SUMMARY OF THE ADVANCE MADE IN THIS PAPER AND ITS POTENTIAL SIGNIFICANCE TO THE FIELD

In this paper Chen et al., use transgenesis approach to test the ability of Eda overexpression to generate ectopic tooth formation in stickleback and zebrafish. I found the paper very well written and in general quite convincing with clear experiments.

SUGGESTIONS TO AUTHORS

However, I have some comments:

1) Throughout the paper the concept of pioneer teeth is used but is never justified and this is not that trivial as it was not easily accepted, I would suggest the authors to explain this explicitly in their introduction. They can use Sadier et al., BioEssays 2020, 1900229 as a source of information. The validity of this concept has been demonstrated in Gibert et al., Proc B 2019 (a paper already cited). Also in the introduction, a general review on Eda signaling could be cited (e.g. Sadier et al., Trends in Genetics, 2014).
2) My main concern is the model linking the expression of the receptors (Edar, Troy and Tret) with the competence. I like the idea, but the current version of the paper does not allow to be fully convinced. First, because given the quality/contrast of Figure 8 it is hard to be fully convinced. Second because then the authors should show that he same receptors are not expressed in the corresponding area in zebrafish as they don't see ectopic teeth in this species outside ceratobranchial. This is easy to check, and I am surprised it has not been done. Third, there are not only the receptors: downstream proteins (e.g. Edaradd etc...) could be important. This should be at least mentioned in the Discussion. On the same aspect I wonder if there are single cell data available in zebrafish or stickleback that could bring useful information at the level?
3) The link between neuromast an extra tooth is interesting but I don't understand why in some case the extra teeth seem to replace the neuromast and in other cases there is juxtaposition. I think the analysis is a bit superficial here and a time series comparing PitX2 expression with a neuromast marker would be useful.
4) The fact that Eda type B in zebrafish induce a much more limited series of extra teeth is interpreted as a difference of competence of pharyngeal areas in both species. Maybe this is correct, but an important control would be to do a Stickleback over expression Zebrafish-EdaB. If this line effectively generates the same pleiade of extra teeth than EdaA then this will be convincing. Otherwise, you cannot exclude a difference of efficiency (stability, partners etc...) between the two proteins.
5) About teeth replacement (and its absence in the ectopic face teeth) it would be interesting to perform in situs with Sox2 a marker of stem cells associated with teeth regeneration. I would expect an absence of Sox2 (hence of stem cells) in the face teeth.
6) The expression of Eda along the midline of cb4 in stickleback is striking. Did the authors look by histology to detect the presence of any weird ectodermal appendage?
7) Figure 5. The labelling of the various bones is not consistent (epi1 for eb1 for example). Where is io?
8) After Figure 7 I think a wrap-up figure would be useful

First revision

Author response to reviewers' comments

© 2025. Published by The Company of Biologists under the terms of the Creative Commons Attribution License (https://creativecommons.org/licenses/by/4.0/).

We have edited our manuscript and figures in accordance with the thoughtful suggestions provided by the reviewers. We modified the text in numerous places, detailed below, and we also described three additional expression assays, including those on *Eda* overexpression sticklebacks (new Fig. S5) and WT zebrafish (Fig. S3D). These new data panels have greatly strengthened the paper. We also created a new summary figure (Fig. S6) at the request of Reviewer 3.

After adding the requested discussion points and references, we additionally streamlined some sections of the discussion to reduce the word count.

Reviewer 1: SUMMARY OF THE ADVANCE MADE IN THIS PAPER AND ITS POTENTIAL SIGNIFICANCE TO THE FIELD

Reviewer 1: This manuscript presents a well-executed study exploring how ectopic overexpression of Ectodysplasin in transgenic sticklebacks and zebrafish can reveal latent tooth-forming competency across multiple cranial and pharyngeal domains. The authors employ temporally controlled heat shock-induced Eda overexpression and demonstrate that ectopic tooth formation is highly spatiotemporally regulated, with pharyngeal teeth exhibiting regenerative potential, while facial teeth do not.

The manuscript is well structured, and includes all necessary details to follow the authors reasoning and experimental pipeline. I did enjoy reading the manuscript. My comments are very minor:

> We thank Reviewer 1 for their overall positive assessment of the manuscript and for their many helpful comments, which we address below.

1. While the study identifies Edar and Troy expression patterns coincident with ectopic tooth formation, functional validation of receptor involvement is lacking. I recommend that the authors acknowledge this as a limitation or suggest it as a direction for future research in their discussion.

> We agree, and have added specific mention of this idea to the discussion section, now starting at line 551:
>
>> "Further tests using Troy and Edar mutants with concurrent Eda overexpression could test whether either of these receptors are necessary for conferring ectopic tooth formation competency. These and other experiments, such as those examining the expression and function of other TNF signaling pathway components, like Edaradd, could additionally test whether the molecular basis of competence for organ formation in this system is defined by differential, or perhaps combinatorial, expression of receptors and associated cellular machinery."

2. The manuscript presents a complex timeline of tooth formation competency without sufficient clarity on the specific developmental windows involved. A clearer presentation of the timing and anatomical specificity would enhance reader understanding.

> We agree. To further clarify the developmental timeline of tooth formation competency revealed by our studies, we have provided a table (Table S1) that outlines specific developmental milestones from 0 to 24 days post fertilization in sticklebacks, highlighting events related to tooth differentiation, both endogenous dental development and ectopic tooth formation in response to heat-shocked Eda. We think this new table should help readers better understand the timing and anatomical specificity of tooth formation competency revealed in this study.

3. The claim that ectopic pharyngeal teeth regenerate autonomously is intriguing but lacks direct evidence of self-renewal without external Eda input. Future studies should use lineage tracing or long-term live imaging to verify that these tooth fields can sustain multiple cycles of tooth replacement. Additionally, pulse-chase experiments or single-cell transcriptomics could provide insights into the cellular mechanisms underlying this regeneration.

> -We agree and have changed the language used in the discussion to explicitly state that the presence of regeneration is a hypothesis rather than a *de facto* conclusion of our data. We

© 2025. Published by The Company of Biologists under the terms of the Creative Commons Attribution License (https://creativecommons.org/licenses/by/4.0/).

clarified that our pulse-chase data supports this hypothesis, but we agree it is not definitive proof of regeneration. We additionally mention that cell lineage tracing would provide additional lines of evidence to support the hypothesis. Excerpt starting at line 414:

> “The ectopic teeth present at these later stages were thus unlikely to have been exposed to exogenous Eda from the OE transgene. Instead, we hypothesize that these later-observed ectopic teeth arise from regeneration events at ectopic tooth fields as part of the typical developmental trajectory of a tooth. Our pulse-chase data in sticklebacks supports this hypothesis, whereby in some cases the arrangement of new and retained teeth was highly consistent with tooth replacement (Fig. 7E’’, arrowhead). Further experiments using cell lineage tracing could determine the origin of these late ectopic teeth testing.”

-We also changed the conclusion (at line 562) to use the phrase “sustained addition of new teeth” rather than “regeneration.”

Reviewer 2: SUMMARY OF THE ADVANCE MADE IN THIS PAPER AND ITS POTENTIAL SIGNIFICANCE TO THE FIELD

The Chen et al is an interesting exploration of how overexpressing Ectodysplasin (Eda) increases tooth number in sticklebacks and zebrafish. What differentiates this study form the previous ones is the construction and use of heat shock-inducible Eda, thereby allowing temporal regulation of Eda overexpression. The results provide a nuanced view into the effects of Eda, most notably how multiple treatments increase the formation of extra teeth in different locations. These suggest sensitivity of tooth making 'potential' being different in different locations of the fish body, many having evolutionary explanations. The analyses and images are high quality and I have no general reservations about the study.

We thank Reviewer 2 for their overall positive assessment of the manuscript and address their many excellent suggestions point-by-point below.

SUGGESTIONS TO AUTHORS

1) It would make the paper stronger if there was a bit more explanation why sticklebacks and zebrafish should be studied in this manner (and not only one of the species). I am not questioning this approach, but as a narrative the reason for the use of these two taxa is left a bit for the imagination of the reader. Yes, zebrafish is the model species, and sticklebacks have more teeth so it is more interesting in this context, or something like that.

We agree that this context is important. We have added a sentence explaining this to the introduction at line 106:

> “By using a comparative approach in two fish species with divergent dental arcades (Square et al., 2021), we can determine whether tooth formation responsiveness demonstrates similar or disparate characteristics among divergent groups.”

2) Presumably the repeated heat shock experiments (very nice Fig 5) increased the expression levels of Eda and availability of the EDA protein. Although measuring expression levels would be difficult in these kind of samples, the Fig 8 expression analyses makes one wonder how some of those would have changed in some of the treated samples (e.g. Fig 8E). I'll leave it for the authors to decide if any additional expression analysis is needed. Presumably the expression domains of Edar and Troy expand before the teeth become visible in the treated samples?

-This is an excellent point worth clarifying: given that Eda is capable of inducing the expression of its own receptors in zebrafish (per Aigler et al., 2014), this is one line of evidence that some other factors beyond a TNFR are required for/participate in the tooth formation response. We have added this thought to the Discussion in the context of pervious zebrafish data at line 546:

© 2025. Published by The Company of Biologists under the terms of the Creative Commons Attribution License (https://creativecommons.org/licenses/by/4.0/).

"[…] zebrafish edar expression in the dorsal pharynx is promoted by Eda OE, suggesting that Eda can somehow upregulate the expression of its own receptor in a region where it was not detected in wild type (Aigler et al., 2014). These data overall strongly suggest that additional factors are required beyond one or more TNFRs to allow ectopic tooth formation in response to Eda.."

-We have additionally added a new Fig. S5 showing that *Edar*, *Troy*, and *Relt* are expressed in early stages of tooth differentiation prior to ossification of ectopic teeth. While the stages we collected evidently capture early tooth germ differentiation rather than stages prior to tooth differentiation (which we cannot observe or confirm prior to ISH), this still does constitute changes to TNFR expression following Eda OE. We describe these in the new Results section at line 372:

"To assess the response of stickleback TNFR expression to Eda OE, we again performed HCR to detect Edar, Troy, and Relt on control and Eda OE larvae. As expected, we found that Edar, Troy, and Relt, which all mark endogenous teeth (Fig. S3), were expressed in nascent ectopic tooth germs on both the face and in the pharynx (Fig. S5). Additionally, we noted that neuromasts in the Eda OE condition (marked by Troy) displayed some disrupted organization relative to the control condition, especially those neuromasts with neighboring early tooth germs (marked by Relt expression)."

3) Line 93 "Provocatively, the ectopic teeth that arose in these experiments were often morphologically discontiguous with endogenous tooth fields,.."

Comment: Not sure what is provocative here. Intriguingly?

This word has been replaced at line 89 per the suggestion here -- we thank the reviewer for pointing this out and agree that "intriguingly" fits better.

4) Line 459: "...while Osr2 loss-of-function has been shown to expand the endogenous molar tooth field in mice.."

Comment: reference?

References have been added to this sentence (now at line 457) – they were previously listed later in this paragraph but we agree they should be here as well.

5) 'Face teeth' is used to describe the ectopic teeth on the stickleback snout. This sounds a bit strange, and one wonders whether 'facial teeth' would be any better. I'll leave it for the authors to decide the nomenclature.

As masks and tattoos on the face are called "face masks" and "face tattoos", we prefer the "face teeth" nomenclature.

6) There is a reference to Fig. S5 in the text.

This should have been "S4" – this has been corrected (now at line 354).

Reviewer 3: SUMMARY OF THE ADVANCE MADE IN THIS PAPER AND ITS POTENTIAL SIGNIFICANCE TO THE FIELD

In this paper Chen et al., use transgenesis approach to test the ability of Eda overexpression to generate ectopic tooth formation in stickleback and zebrafish. I found the paper very well written and in general quite convincing with clear experiments.

We thank Reviewer 3 for their overall positive assessment of the manuscript. Below we address their many thoughtful comments in detail.

© 2025. Published by The Company of Biologists under the terms of the Creative Commons Attribution License (https://creativecommons.org/licenses/by/4.0/).

SUGGESTIONS TO AUTHORS

However, I have some comments:

1) Throughout the paper the concept of pioneer teeth is used but is never justified and this is not that trivial as it was not easily accepted, I would suggest the authors to explain this explicitly in their introduction. They can use Sadier et al., BioEssays 2020, 1900229 as a source of information. The validity of this concept has been demonstrated in Gibert et al., Proc B 2019 (a paper already cited). Also in the introduction, a general review on Eda signaling could be cited (e.g. Sadier et al., Trends in Genetics, 2014).

> -We agree and now better define pioneer teeth in the introduction at line 118 and cite the first requested reference there:
>
> > "We further find that zebrafish and sticklebacks both demonstrate the highest potential to form ectopic pharyngeal teeth during the window wherein their first endogenous teeth (known as "pioneer teeth") are specified, which are hypothesized to possess special characteristics as compared to other endogenous primary teeth (Gibert et al., 2019; Sadier et al., 2020)."
>
> -We added the second requested reference at line 75:
>
> > "Once bound by Eda, Edar activates the Nuclear Factor Kappa B (NF-κB) pathway which induces transcription of genes involved in epithelial organ specification, including teeth (Liang et al., 2019; Ohazama and Sharpe, 2004; Sadier et al., 2014; Schmidt-Ullrich et al., 2006)."

2) My main concern is the model linking the expression of the receptors (Edar, Troy and Tret) with the competence. I like the idea, but the current version of the paper does not allow to be fully convinced. First, because given the quality/contrast of Figure 8 it is hard to be fully convinced. Second because then the authors should show that he same receptors are not expressed in the corresponding area in zebrafish as they don't see ectopic teeth in this species outside ceratobranchial. This is easy to check, and I am surprised it has not been done. Third, there are not only the receptors: downstream proteins (e.g. Edaradd etc...) could be important. This should be at least mentioned in the Discussion. On the same aspect I wonder if there are single cell data available in zebrafish or stickleback that could bring useful information at the level?

> -We have adjusted contrast in Fig. 8A', A", A'''', and B to further clarify these expression data.
>
> -We thank the reviewer for the thoughtful suggestion regarding TNFR expression in zebrafish. We have added a panel to Fig. S3 with zebrafish expression of *edar* and *troy* (Fig. S3D). This panel shows that *edar* is not detected in ceratobranchials 1-4 at 2 dpf as it is in sticklebacks at 6 dpf. Admittedly, we are unable to resolve *edar* and *troy* expression in zebrafish at the resolution we can achieve on the much larger sticklebacks. We added the following summary to the Results section at line 355:
>
> > "To ask whether zebrafish also express edar and troy in their ventral pharynx despite lacking a tooth formation response in these regions under eda OE, we also assayed the expression of these receptors via HCR at 2 dpf, the timepoint we found to have the highest rate of ectopic tooth formation. We found that troy was expressed throughout the ventral pharynx, similar to our observations of stickleback Troy at 6 dpf, but edar was not detected in this same region as it was in sticklebacks (Fig. S3D). We additionally detected both TNFRs in the pioneer tooth germ at this stage (black arrowhead in Fig. S3D)."
>
> -We additionally expanded and rewrote the corresponding Discussion section related to the association between ectopic tooth formation and TNFR expression (also related to requests by both other Reviewers), starting at line 536:

© 2025. Published by The Company of Biologists under the terms of the Creative Commons Attribution License (https://creativecommons.org/licenses/by/4.0/).

"Despite the associations we observed between TNFR expression and ectopic tooth specification, other factors are clearly required for the ectopic tooth formation response. First, we detected Edar or Troy expression in other stickleback tissues that never formed ectopic teeth in our experiments (such as gill and gill raker precursors, respectively). Second, publicly available single cell RNA-seq data suggests that zebrafish neuromasts likely express troy at 5 dpf (Lange et al., 2023), and morpholino injections targeting troy interfered with proper neuromast differentiation (Han et al., 2014), suggesting that troy-positive neuromast cells are not necessarily competent to form teeth under Eda OE in zebrafish. Third, zebrafish edar expression has been previously shown in epithelia overlying the dentary and premaxilla, where ectopic tooth formation is not triggered by Eda OE (Aigler et al., 2014). Fourth, zebrafish edar expression in the dorsal pharynx is promoted by Eda OE, suggesting that Eda can somehow upregulate the expression of its own receptor in a region where it was not detected in wild type (Aigler et al., 2014). These data overall strongly suggest that additional factors are required beyond one or more TNFRs to allow ectopic tooth formation in response to Eda. Further tests using Troy and Edar mutants with concurrent Eda overexpression could test whether either of these receptors are necessary for conferring ectopic tooth formation competency. These and other experiments, such as those examining the expression and function of other TNF signaling pathway components, like Edaradd, could additionally test whether the molecular basis of competence for organ formation in this system is defined by differential, or perhaps combinatorial, expression of receptors and associated cellular machinery."

-We have added specific mention of Edaradd in this context and brought up the possible roles for non-receptor machinery (at line 555, within the excerpt pasted above).

-We assessed publicly available single cell data both prior to submitting and again following the reviewer's suggestion here. Currently published zebrafish single cell sequencing does not readily provide meaningful insights as to which cells arose from the dorsal pharynx, anterior to the keratinous chewing pad (the only location of ectopic tooth formation in zebrafish), or reveal which cells might be derived from cb4 at 2 dpf. However we are able to see that *troy* is likely expressed in zebrafish neuromasts at 5 dpf. We have added mention of this to the discussion and cited the publicly available single cell data (at line 540, within the excerpt pasted above).

3) The link between neuromast an extra tooth is interesting but I don't understand why in some case the extra teeth seem to replace the neuromast and in other cases there is juxtaposition. I think the analysis is a bit superficial here and a time series comparing PitX2 expression with a neuromast marker would be useful.

-We agree with the reviewer that the spatial location of extra teeth (e.g. whether they replace neuromasts or form juxtaposed to neuromasts) is an important detail. We have clarified that juxtaposition is the typical pattern on line 209:

"Thus, ectopic teeth on the outside of the face are highly spatially biased, typically forming juxtaposed to neuromasts, and occasionally in the place of neuromasts, suggesting copatterning between neuromasts and Eda-driven ectopic teeth."

-A similar request was made by Reviewer 2 with respect to data on ectopic tooth germ gene expression (Rev. 2 item 2); we believe these suggestions have strengthened our manuscript. To address both of these requests, we have included HCR on control vs Eda OE fish faces (New Fig. S5B), showing expression of *Troy* (neuromast marker) alongside *Edar* and *Relt* (tooth germ markers). New results are described at line 372 (see Rev 2 item 2 for pasted excerpt).

4) The fact that Eda type B in zebrafish induce a much more limited series of extra teeth is interpreted as a difference of competence of pharyngeal areas in both species. Maybe this is correct, but an important control would be to do a Stickleback over expression Zebrafish-EdaB. If this line effectively generates the same pleiade of extra teeth than EdaA then this will be

© 2025. Published by The Company of Biologists under the terms of the Creative Commons Attribution License (https://creativecommons.org/licenses/by/4.0/).

convincing. Otherwise, you cannot exclude a difference of efficiency (stability, partners etc...) between the two proteins.

We agree that this possibility regarding potential differences in the effects of zebrafish and stickleback Eda proteins should be explicitly stated and discussed -- we added mention of this alternative explanation in the Discussion section at line 495:

"Whether differences in the amino acid sequence of Eda proteins underlie these dental competency differences is unknown, which could be tested by overexpressing Eda in a cross-species manner (e.g. overexpression of stickleback Eda in zebrafish). Notably, overexpression of zebrafish eda in Astyanax caused ectopic teeth to form in the ventral pharynx (Jandzik and Stock, 2021), suggesting that not all observed differences in dental competency across species are due to Eda amino acid sequence."

5) About teeth replacement (and its absence in the ectopic face teeth) it would be interesting to perform in situs with Sox2 a marker of stem cells associated with teeth regeneration. I would expect an absence of Sox2 (hence of stem cells) in the face teeth.

We agree with the reviewer. We previously mentioned this idea in the Discussion section, but we have now augmented this sentence with the names of specific genes, including Sox2 at line 434:

"Further analyses of gene expression associated with successional dental cell types, such as Sox2, Lgr6, and Nfatc1 (Juuri et al., 2012; Martin et al., 2016; Rasch et al., 2016; Square et al., 2021) and additional experiments with longer observation windows could resolve these possibilities."

6) The expression of Eda along the midline of cb4 in stickleback is striking. Did the authors look by histology to detect the presence of any weird ectodermal appendage?

We agree that the expression of Eda along the midline of cb4 is interesting given that no endogenous teeth normally form in this region. We have observed no epithelial ornaments there besides gill rakers (nearby laterally) and taste buds (towards the lumen), making this region composed of nothing different than the serially homologous regions found more anteriorly (e.g. hb1 and hb2) which do not show *Eda* expression by our assays. We have added mention of this point to the discussion at line 406:

"We observed no epithelial appendages in this region of cb4, leaving the potential function (if any) of this Eda expression domain a mystery."

7) Figure 5. The labelling of the various bones is not consistent (epi1 for eb1 for example). Where is io?

-We thank the reviewer for catching this inconsistency regarding epibranchial 1 abbreviations, it has been corrected in the newly uploaded Fig. 5.

-We have added mention of where to see the position of the io in context to the Figure legend (line 791: "See Figs. 2 and 3 for io position in context.").

8) After Figure 7 I think a wrap-up figure would be useful

We have added a new wrap-up figure to the manuscript (new Fig. S6) to summarize the regions of tooth formation competency as they relate to TNFR gene expression.

Second decision letter

© 2025. Published by The Company of Biologists under the terms of the Creative Commons Attribution License (https://creativecommons.org/licenses/by/4.0/).

MS ID#: dev.204907R1

MS Title: Ectodysplasin overexpression reveals spatiotemporally dynamic tooth formation competency in stickleback and zebrafish

Authors: Zoe Z. Chen; Sujanya N. Narayanan; Lena M. Stagliano; Peter Q. Huynh; Shivani Sundaram; Emma J. Mackey; Craig T. Miller; Tyler A. Square
Article Type: Research Article

Dear Tyler,

I am happy to tell you that the referees are happy with your revisions and your manuscript has been accepted for publication in Development, pending our standard publication integrity checks

Reviewer 1

Many thanks to the authors for their thorough revision and for addressing my questions. I can now recommend the manuscript for publication.

Reviewer 3

The authors have handled the revisions sensitively. The new version is fully acceptable. This is a really cool story. Congratulations.

© 2025. Published by The Company of Biologists under the terms of the Creative Commons Attribution License (https://creativecommons.org/licenses/by/4.0/).
